# Informing future quarantine practices through the experiences of COVID‐19 quarantine facility staff

**DOI:** 10.1002/puh2.184

**Published:** 2024-05-29

**Authors:** Angela Sheedy, Dianne Stephens, Lisa Vermeulen

**Affiliations:** ^1^ CDU Menzies School of Medicine Charles Darwin University Darwin Australia

**Keywords:** COVID‐19, health care workers, pandemic, quarantine, survey

## Abstract

**Background:**

The COVID‐19 pandemic necessitated the rapid development of quarantine sites, prompting the need for new staff models and scopes of practice. This project surveyed health and non‐health staff at a large outdoor quarantine facility in regional Australia to gather insights for future quarantine facility guidelines based on their experiences and perceptions.

**Methods:**

This translational research project implemented a mixed‐methods approach to analyse staff perceptions of working at a quarantine facility to inform the development of a policy and practice guide. An anonymous online survey utilising purposive sampling was distributed to 410 multidisciplinary survey participants over an 8‐week period. Survey questions focussed on site processes, challenges and recommendations for future implementation of quarantine services. Qualitative data was thematically analysed with the aid of Leximancer, and a descriptive statistical method was used for quantitative data analysis.

**Results:**

There were 92 survey respondents from health and non‐health roles; of these, 85% indicated they would work at the quarantine facility again, and 90% agreed residents were well cared for. There was a lack of anxiety of acquiring COVID‐19, with 95% feeling safe from COVID‐19 transmission onsite. Challenges staff identified highlighted future investment areas, including leadership communication models, information technology (IT) management systems specific for quarantine services and site processes to better accommodate weather elements.

**Conclusion:**

Overall, staff validated the primary health model of quarantine care with key challenges highlighting the importance of leadership and investment in communication and IT. The results were aligned with site functions and operations and will inform the development of a pandemic quarantine facility guide.

## Graphical abstract

The COVID‐19 pandemic necessitated the establishment of quarantine facilities; a survey at a large outdoor quarantine facility in Australia gathered insights from multidisciplinary staff. The findings emphasised the importance of leadership, communication and information technology in managing quarantine services with staff validating the facility's primary health model and indicating a willingness to work there again.

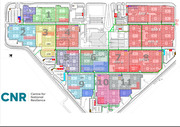

## INTRODUCTION

The COVID‐19 pandemic required extraordinary and evolving public health measures and included the widespread use of quarantine facilities. Effective quarantine approaches can slow infection rates, allow governments time to prepare health systems and resources and ramp up a surge response workforce. In Australia, quarantine was delivered in a variety of models with the Centre for National Resilience (CNR) quarantine facility in the Northern Territory (NT) held out to be the gold standard, presenting an exceptional record of zero transmission from the facility into the community during its period of operation. The health model of quarantine care at CNR differed from other quarantine models by the site being outdoors, implementing foundational primary health services and being led by the Department of Health, governed by public health principles and supported by the Northern Territory Government (NTG) and non‐government organisations [1, 2].

Across the globe, the implementation of quarantine was varied, from special health accommodation to health hotels, ships and purpose‐built or adapted facilities, all encountering different challenges. Qatar, for example, presented an industrial‐scale quarantine model that involved acquiring suitable premises and redeveloping them for quarantine, whereas in Pakistan, a large rehabilitation facility was converted and overseen by the Pakistani military [3, 4, 5]. Europe and the Americas repurposed buildings, and Australia and New Zealand used a mix of hotel and government‐managed facilities. Overall, the shared goal of the quarantine facility was to prevent virus spread and reduce the burden on the wider health system.

With a number of incidences in Australian hotel quarantine causing widespread community outbreaks, lessons learned emphasise that quarantine services must be safe for both quarantine workers and residents, cost‐effective and resource‐efficient [2, 6]. The health‐led model of quarantine at CNR incorporated a resident‐centred care model involving daily health checks with onsite access to basic primary care and a referral system to acute health care services. There was extensive infection prevention and control (IPC) training for all staff and an evolving set of practices responsive to disease trends.

**FIGURE 1:**
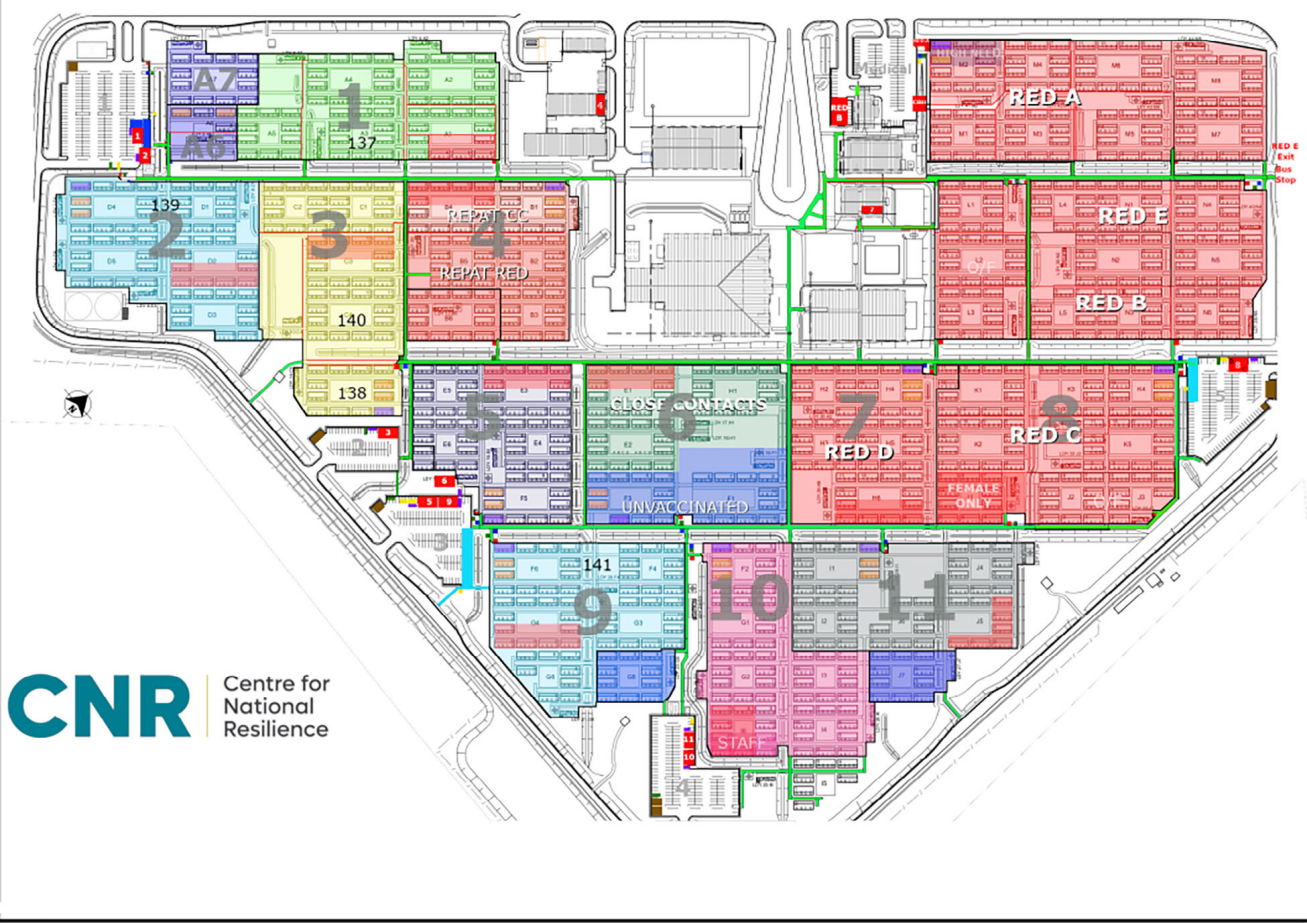
Map of Centre for National Resilience, Howard Springs Quarantine Facility, presenting resident zones (coloured areas 1–11), the red zone used for positive COVID‐19 cases (red A–E) and staff areas (uncoloured areas).

Located in the tropical north of Australia, 25 km from Darwin in the NT, Australia, CNR was originally a workers’ camp adapted for quarantine. It served as a role model for similar services, hosting over 2000 residents daily, including domestic, international and repatriated individuals [7]. The site began operations early in the pandemic (February 2020) and followed public health quarantine principles, using zones to logistically segregate infected (red zone), potentially infected (orange zone) and not infected people (green zones) (refer to Figure 1). Residents were additionally cohorted according to their flights, family status and COVID‐19 results.

Staffing a large facility in this remote region of Australia was challenging and required innovative approaches. The resulting resident‐facing workforce included a multidisciplinary team of health professionals and skilled and unskilled workers. The diverse unskilled workforce was rapidly recruited, providing employment at a critical time in the pandemic and vital to ensure the existing acute and primary health systems remained staffed, a strategy utilised by other jurisdictions [8, 9]. New approaches in quarantine staffing included the Assistant in Nursing (AIN) programme which utilises second‐ and third‐year undergraduate nursing students in health service provision [10]. Non‐health workers were employed in administration roles to work alongside the professional health staff (nurses, physiotherapist and medical officers), contributing to the direct face‐to‐face and behind‐the‐scenes resident care, a model not dissimilar to the use of community health workers in countries where registered health professionals are scarce [11]. The rapid inclusion of this new administration officer role required a novel scope of practice definition and demonstrated how versatile a workforce can become when pushed by emergency requirements to protect the greater community.

A resilient pandemic workforce requires systemic support, education and training about the risks in the workplace with access to the resources to protect them from disease transmission [12]. Fear of COVID‐19 disease transmission was experienced globally by many health workers, as well as depression, anxiety and stress associated with working in a COVID‐19 frontline position [13, 14, 15]. Many frontline workers across the world experienced stigma for working directly with COVID‐19‐positive people, confronted with negative community perceptions associated with fear of disease transmission from quarantine staff [16]. Such evidence highlights the importance of quarantine facilities providing a COVID‐19‐safe environment with systems in place to protect and support staff experiencing stress or anxiety.

This research evaluated the CNR quarantine model through an online survey of staff experiences and perceptions and focussed on: site processes and infrastructure, IPC, resident management, challenges and recommendations for change. The project specifically aimed to learn from staff feedback to inform the development of a pandemic quarantine facility toolbox. Recognising that the establishment of quarantine services was new and rapid during COVID‐19 with many lessons now learned, this toolbox aims to present coherent and evidence‐based guides for future use in quarantine and isolation of individuals and populations.

## METHODS

This translational research project implemented a mixed‐methods approach to analyse staff perceptions of working at the quarantine facility to inform the development of a quarantine facility policy and practice guide. Translational research in public health actions such as with quarantine services seeks to enhance population health by fostering evidence‐based policy and practice [17].

### Quarantine staff survey development

An anonymous online Qualtrics survey with implied participant consent via the use of an opt‐out option was distributed by email to the functional work groups contributing to the day‐to‐day service provision for onsite resident care. A project team workshop was conducted with two senior nursing staff, the director of logistics and a senior medical professor who had worked onsite to develop the survey questions. There was no pilot testing of the survey, and validation was completed in the project team workshop, where survey questions were critiqued for incomplete or inaccurate question structure and wording problems, with a focus on ensuring the scale applied to the questions was consistent and measured what was intended. Open and closed survey questions were based on four themes that correlated with core quarantine functions: site infrastructure, site processes (including communication), IPC practices and resident care (resident entry, management and exiting the facility). The survey included additional questions focussed on staff roles, challenges they feel they encountered whilst carrying out their duties, and their recommendations in the event the quarantine service was required to recommence service provision. A separate survey question set was inserted for staff who identified as health professionals to direct them to questions on the provision of resident primary health (clinical) care. These questions omitted security, cleaning or catering staff.

Closed response questions implemented a Likert scale and grouped questions to maintain internal consistency, offering options to strongly agree, somewhat agree, neither agree nor disagree, somewhat disagree or strongly disagree with a not applicable option. The not applicable option was deemed necessary to accommodate staff from areas such as cleaning and catering who had limited or no involvement in aspects of the quarantine service processes.

### Participant recruitment

A sample size of 410 multidisciplinary survey participants was targeted, noting that the population size of quarantine workers fluctuated onsite due to general staff attrition. The target size of 410 was based on workers present at or around the time the quarantine site closed. Participants from government organisations were contacted with permission from the Department of Health via their professional emails, and the directors of non‐government organisations were recruited to send the email to their staff members. All participants received the same email information and link to the same survey. The survey was delivered using Qualtrics and remained open for responses for 8 weeks.

### Data analysis

A mixed‐methods approach was used for data analysis. Thematic analysis was implemented with primary data (open‐question responses) aided by Leximancer (a validated tool for qualitative analysis). Survey responses were both manually analysed to identify core trends in feedback and through Leximancer text analysis for further recognition of concept occurrence/frequency and concept mapping. Quantitative data analysis utilised a descriptive statistical method to identify response trends; this was enhanced by Qualtrics survey reporting with the use of question matrix functions and scaling.

### Ethical consideration

This project received ethics review and approval from the Human Research Ethics Committee of the Northern Territory Department of Health and Menzies School of Health Research (HREC Number 2022‐4349). Participants were provided with a research plain language statement; participation was voluntary with an opt‐out approach to the survey.

## RESULTS

The survey had 92 respondents (a response rate of 22%); the response rate was lower than anticipated likely due to the survey being offered post‐closure of the facility, resulting in participants being less invested and interested in providing feedback. The separate survey question set for staff who identified as health professionals and government workers included 66 of the original 92 survey participants, with 26 security, cleaning and catering staff not having access to these questions.

### Staff roles

The survey found that 41% of participants were health professionals and 59% were non‐health staff, with 28% from non‐governmental organisations (as presented in Table 1). All worked with NT domestic residents, international arrivals, Commonwealth repatriation and humanitarian flights as part of the NT remote community response, and 84.5% of respondents worked in COVID‐19‐positive zones, indicating a high level of risk environment. Rapid recruitment strategies were considered effective, with 78% (58 of 73 *responses*) agreeing they ended up in the job they applied for. Regarding orientation, 88% felt adequately prepared, and 92% reported reduced anxiety after completing orientation. Staff highlighted teamwork as a key aspect of working at CNR, rating their employment experience positively (refer to Table 2). Areas for improvement included workload equity, staff wellbeing and education.

**TABLE 1:** Breakdown of Centre for National Resilience, Howard Springs Quarantine Facility staff survey respondents demonstrating the staff mix of health and non‐health professionals.

Staff area of practice	Number of participants *n* (%)
Nursing (including registered, enrolled and student nurses)	34 (39.6)
Administration officer	17 (17)
Aboriginal engagement team	3 (3)
Medical officer	4 (4)
Allied health (includes interpreters, social work, psychologists and physiotherapist)	0 (0)
TeleWellbeing/Tele Health and Health welfare tam	6 (6)
Operations/Ground support	2 (2)
Security	9 (9)
Cleaning staff	11 (11)
Catering staff	6 (6)
Total	92*N*

**TABLE 2:** Centre for National Resilience (CNR) staff survey question set focussed on staff roles and experience (*N* = 92).

							Qualtrics measured		
Staff roles and experience: How would you rate your employment at CNR	Strongly agree (%)	Somewhat agree (%)	Neither agree nor disagree (%)	Somewhat disagree (%)	Strongly disagreed (%)	Not applicable (%)	Standard deviation	Variance	Response *n* (%) (92*N*)
I would have stayed longer if given the opportunity	51 (66.5)	11 (14.5)	5 (6)	5 (6)	3 (4)	2 (3)	1	2	77 (84)
I would work at a quarantine/isolation facility again.	56 (74)	16 (21)	2 (3)	1 (1)	1 (1)	0 (0)	1	1	76 (82)
I was provided the opportunity of professional development	40 (54)	17 (23)	6 (8)	5 (7)	4 (5)	2 (3)	1	2	74 (80)
Working at CNR provided me with new skills that will be useful for my future career	47 (63)	16 (22)	5 (7)	3 (4)	2 (3)	1 (1)	1	1	74 (80)

### Site processes

Survey questions regarding site processes included communication, and these were directed at health and government staff only (excluding security, cleaning and catering staff as they did not have access to the onsite government communication systems). Communication questions revealed 96% (48 of 50 *responses*) agreed they received regular site updates, which demonstrated staff used the communication systems in place; however, only 54% (26 of 49 *responses*) valued the site newsletter (refer to Table 3). The open question set asking *What do you think would improve communication practices onsite for staff?* presented requirements to develop a clear hierarchical communication structure with a reactive response cycle and one information technology (IT) system for site management. This correlated with the open question set on site processes, which highlighted that the use of multiple IT management systems (ITMS) across resident and site management was problematic for staff.

**TABLE 3:** Centre for National Resilience staff survey question set focussed on communications methods onsite for health staff (*N* = 66).

							Qualtrics measured		
Communication methods on site for health staff^a^	Strongly agree (%)	Somewhat agree (%)	Neither agree nor disagree (%)	Somewhat disagree (%)	Strongly disagree (%)	Not applicable (%)	Standard deviation	Variance	Response *n* (%) (66*N*)
I received regular updates on COVID‐19 and changes in the CHO directions	38 (76)	10 (20)	0 (0)	1 (2)	0 (0)	1 (2)	1	1	50 (76)
I used the online TEAMS site to keep up to date with important site news	23 (46)	14 (28)	4 (8)	3 (6)	2 (4)	4 (8)	2	2	50 (76)
I valued the fortnightly newsletter	14 (29)	12 (25)	11 (22)	0 (0)	3 (6)	9 (18)	2	3	49 (74)
I used the RISKMAN system for reporting incidents and accidents onsite	25 (51)	8 (16)	2 (4)	0 (0)	1 (2)	13 (27)	2	5	49 (74)
I received notification of the education and training sessions being held onsite	29 (61)	11 (23)	2 (4)	4 (8)	0 (0)	2 (4)	1	2	48 (72)
The monthly all staff site meeting kept me informed	25 (51)	9 (18)	4 (9)	2 (4)	1 (2)	8 (16)	2	3	49 (74)

^a^ Question set only provided to NTG and health staff, excluded catering, cleaning and security staff.

The closed question set on the site mandatory staff swabbing (viral screening) and the COVID‐19 vaccination process demonstrated all 75 respondents understood the site's COVID‐19 swabbing policy, and 98% (74 respondents) received information on the vaccination process, with 97% (73 of 76 *responses*) understanding the vaccination requirement.

### Site infrastructure

The survey questions on the site infrastructure related to the physical layout of the quarantine service were directed at all staff. Overall, 93% (67 of 72 responses) agreed the site worked well, but fewer agreed on the sectioning and fencing of resident zones (78%, 57 of 73 responses) and that the general zone layout (75%, 56 of 74 responses) worked well. A total of 61% (45 of 73 responses) agreed that the size and layout of the facility did not affect their work practices.

The main concept to emerge from the open‐answer question *What do you think could improve the layout and management of HSQF for staff?* was based on the need for staff self‐responsibility with their health and safety with environmental safety. An additional question set focussed on weather and heat challenges due to the sites open plan was included, with time, heat, distance and PPE identified as key challenging environmental considerations. Staff felt adequately informed about storm warnings (91%, 66 of 72 agreeance) and understood wet weather's impact on PPE (98%, 71 of 72 agreeance).

### Infection prevention and control

The quarantine site practices prioritised IPC, with 95% of staff feeling safe from COVID‐19 transmission onsite and 94% endorsing the effectiveness of the buddy system. However, satisfaction with IPC practices varied, with 81% satisfied with donning and doffing stations and 88% observing safe practices, among others. Only 75% (54 of 72 responses) agreed that having security based at the donning and doffing station contributed to their safety in the zone area. Staff provided 28 comments for the open‐answer question, *Please provide any additional personal observations or commentary about infection prevention measures at HSQF*. The main themes to emerge were concerning staff behaviours, coherent site policies and practices and poor resident behaviours, PPE resources and the buddy system, and having the security guard posted at the donning and doffing station for IPC supervision. Critical appraisal focussed on staff behaviours related to breaches in IPC practice, and this centred on non‐health staff (such as police, security and cleaners).

### Resident care

The response rate for resident care questions was lower (around 50 responses per question) due to restricted question access for security guards, cleaners and catering staff. Overall, approaches to manage resident arrival and exit were successful (refer to Table 4). Staff agreed residents were well cared for, with 90% valuing the face‐to‐face visits by health staff for residents’ health and wellbeing and praising the open‐air balcony model. Area staff ranked lower related to residents’ access to health services and acute care management. Higher levels of staff disagreed with the site process (between 8% and 10%) pertaining to resident referrals, access to allied health services, children in isolation and acute care management. Open‐answer responses highlighted staff felt further opportunities for physical activity for residents would be beneficial with two core themes: better communication and health and wellbeing systems.

**TABLE 4:** Centre for National Resilience staff survey question set focussed on resident arrivals, care and management and resident exits (*N* = 66).

							Qualtrics measured		
Resident care and management whilst onsite^a^	Strongly agree (%)	Somewhat agree (%)	Neither agree nor disagree (%)	Somewhat disagree (%)	Strongly disagree (%)	Not applicable (%)	Standard deviation	Variance	Responses *n* (%) (66*N*)
Face‐to‐face visits by the health team were important for resident health and wellbeing	44 (88)	3 (6)	1 (2)	0 (0)	0 (0)	2 (4)	1	1	50 (75)
The Telewellbeing team was important for resident health and wellbeing	38 (74)	6 (12)	4 (8)	1 (2)	0 (0)	2 (4)	1	1	51 (77)
The residents were well supported at HSQF	29 (58)	16 (32)	2 (4)	1 (2)	0 (0)	2 (4)	1	1	50 (75)
I was able to provide the residents with the care they needed	23 (47)	18 (37)	5 (10)	1 (2)	0 (0)	2 (4)	1	1	49 (74)
The referral system for residents to specialist areas worked well (i.e.: doctors, social work)	21 (41)	20 (39)	3 (6)	3 (6)	2 (4)	2 (4)	1	2	51 (77)
Residents had access to allied health services as they needed	19 (39)	16 (33)	6 (12)	5 (10)	0 (0)	3 (6)	1	2	49 (74)
Children in isolation/quarantine were well supported	22 (44)	18 (36)	5 (10)	2 (4)	1 (2)	2 (4)	1	2	50 (75)
Residents requiring acute care were managed efficiently	24 (48)	14 (28)	6 (12)	4 (8)	0 (0)	2 (4)	1	2	50 (75)
The rooms were comfortable for residents	27 (54)	19 (38)	1 (2)	2 (4)	0 (0)	1 (2)	1	1	50 (75)
Having a balcony/open‐air access was a positive part of the quarantine set up for residents	47 (94)	1 (2)	0 (0)	0 (0)	1 (2)	1 (2)	1	1	50 (75)
The resident handbook was a good resource for residents	34 (68)	9 (18)	3 (6)	1 (2)	1 (2)	2 (4)	1	2	50 (75)
Resident who became COVID‐19 positive or symptomatic onsite were managed safely	33 (66)	14 (28)	1 (2)	0 (0)	0 (0)	2 (4)	1	1	50 (75)
I observed staff to be compassionate and caring of residents wellbeing	35 (70)	11 (22)	2 (4)	0 (0)	0 (0)	2 (4)	1	1	50 (75)

**Resident site arrivals** ^a^	Strongly agree (%)	Somewhat agree (%)	Neither agree nor disagree (%)	Somewhat disagree (%)	Strongly disagree (%)	Not applicable	Standard deviation	Variance	Responses *n* (%) (66*N*)
Planned resident arrivals by bus onsite were streamlined and uncomplicated for residents	26 (52)	15 (30)	3 (6)	1 (2)	3 (6)	2 (4)	1	2	50 (75)
Small group arrivals (10 people or less) on site were streamlined and uncomplicated for residents	24 (49)	19 (39)	1 (2)	2 (4)	1 (2)	2 (4)	1	1	49 (74)
Planned residents arrivals by bus onsite were streamlined and uncomplicated for staff	23 (47)	18 (37)	2 (4)	2 (4)	2 (4)	2 (4)	1	2	49 (74)
Small group arrivals (10 people or less) on site were streamlined and uncomplicated for staff	22 (45)	20 (41)	1 (2)	2 (4)	2 (4)	2 (4)	1	2	49 (74)
As a staff member, I was well informed of my role for resident arrivals	30 (60)	12 (24)	4 (8)	2 (4)	1 (2)	1 (2)	1	1	50 (75)
Residents were well informed of the rules of the quarantine and isolation facility on arrival	26 (53)	10 (21)	4 (8)	5 (10)	1 (2)	3 (6)	1	2	49 (74)

							Qualtrics measured		
**Resident site exits** ^a^	Strongly agree (%)	Somewhat agree (%)	Neither agree nor disagree (%)	Somewhat disagree (%)	Strongly disagree (%)	Not applicable (%)	Standard deviation	Variance	Responses *n* (%) (66*N*)
Planned resident exits by bus onsite were streamlined and uncomplicated for residents	20 (39)	21 (41)	3 (6)	3 (6)	2 (4)	2 (4)	1	2	51 (77)
Small group exits (10 people or less) on site were streamlined and uncomplicated for residents	21 (41)	20 (39)	2 (4)	4 (8)	2 (4)	2 (4)	1	2	51 (77)
Planned resident exits by bus onsite were streamlined and uncomplicated for staff	22 (43)	16 (31)	3 (6)	3 (6)	5 (10)	2 (4)	1	2	51 (77)
Small group exits (10 people or less) on site were streamlined and uncomplicated for staff	23 (45)	15 (27)	4 (8)	4 (8)	3 (6)	2 (4)	1	2	51 (77)
As a staff member, I was well informed of my role for resident exits	28 (54)	15 (29)	2 (4)	2 (4)	1 (2)	3 (6)	1	2	51 (77)

^a^ Question set only provided to NTG and health staff, excluded catering, cleaning and security staff.

### Challenges and recommendations

In the final survey questions, staff were asked, *What did you find most challenging when working at the quarantine service?* There were 62 responses and results linked back to known areas such as challenges with the weather and heat, as well as highlighting less visible challenges such as working with inexperienced staff and dealing with the initial fear of the virus. The main challenges were divided into three concepts:

Internal factors which are under the control of the service: work expectations, communication and site operations.

Factors both internal and external to service control: staff behaviours and residents.

External factors that are not controlled by the service: weather, government legislation and CHO Directions.

There were 57 open responses to the question, *What would you like to see changed if we needed to open the quarantine facility again?* with analysis showing communication as a core theme, predominantly associated with leadership, presence and reliability (refer to Table 5).

**TABLE 5:** Open survey response analysis for staff recommendations for Centre of National Resilience site operations on the reopening of quarantine services.

Core themes	Descriptive themes	Relevant staff survey quotes
Leadership	Communication, presence and visibility	*Better opportunities and avenues for staff to voice concerns or provide feedback to management* *Public facing information about the facility to ensure people knew what to expect and celebrated the work being done*
Site operations	Infection prevention and control, information technology (IT), buggies (vehicles to mobilise across site), logistics, weather	*More supervision or random checks on staff donning/doffing and proper use of PPE whilst walking through the zone* *Radios available from the beginning* *More control of room keys for health staff doing the intakes and a live map of rooms that are unavailable. And who is in each room. Live updates. Controlled by one dept*.
Staff Factors	Pay, promotion, staff‐to‐resident ratios, accountability, education and training	*Better roster system that met resident needs* *Provide better pay rate because it is so hard to work in a such hot weather* *More training opportunities to keep nursing skills relevant and up‐to‐date*
Resident factors	Resident management	*Residents being allowed to move and walk more freely* *Perhaps, firmer communication with some international flights, compliance was an issue at times* *More resident interaction. When TeleWellbeing was running, it meant that every resident was contacted throughout their stay; this was so important because relationships were built and the residents knew they had someone at the end of the phone they could speak to. It also took pressure off the nursing staff on site to go and visit them face‐to‐face*

Staff felt investment in the following areas was required:

‐ Leadership communication, presence and visibility.
‐ Site operations regarding purpose‐designed ITMS systems.
‐ Staff promotion opportunities, salaries, staff‐to‐resident ratios, education and training.
‐ Resident management and communication with more access to physical activity.

## DISCUSSION

Building health workforce capacity is a global focus area with a current deficit of over fifteen million health workers, and therefore understanding how a large quarantine service operated in regards to staffing can inform future health workforce directions. Rapid recruitment and new team models at CNR involved health and non‐health workers collaborating on resident care, presenting successful workforce capacity enhancement. This approach suggests that non‐health staff can be effectively integrated into pandemic teams; however, staff preparation is vital for this workforce model. At CNR, preparation included site orientation, introducing site structures, staff support services, COVID‐19 staff and resident‐specific requirements (such as viral screening) and IPC practices. Survey responses demonstrated that staff felt well prepared for their role, and this highlights how important orientation can be for future quarantine service investment.

Staff ratings indicated high satisfaction with their work, and this is in paradox to the often‐negative work reflections of other frontline COVID‐19 workers [18, 19]. This is particularly interesting given that the results also indicated the majority of staff were working in a high‐risk COVID‐19 environment and yet felt safe from disease transmission. There could be several factors contributing to this; however, it is likely due to structured and inclusive orientation, ongoing education and training, Standard Operating Procedures plus the structure of the buddy system, which meant non‐health staff were always supported by health professionals when working in direct contact or conducting a high‐risk activity with residents.

During COVID‐19, frontline staff often faced changes in site strategies as disease trends redirected priorities and policies, acknowledging that the pandemic led to a COVID‐19 infodemic [8, 20]. Clear communication onsite was one area flagged as requiring more investment, particularly in regard to senior staff support and requests for a reactive communication framework (a common theme across all survey feedback). This aligns with other frontline worker research (in COVID‐19 and previous pandemics) highlighting the importance of investment in resources and strategies for clear communication from leadership teams [21, 22].

Outcomes from previous pandemic responses such as the H1N1 made recommendations for investment in ITMS, yet it appears little progressed in regional areas [23, 24]. Current research identifies ITMS as a common early pandemic problem with systems gradually expanding to adapt to COVID‐19 health and quarantine management requirements [8, 25]. This was also problematic at CNR, with ITMS systems hastily established or adapted for use across resident health and management, Telewellbeing, site maintenance, contractors and site operations. The staff survey responses consistently indicated this was an area requiring investment for future quarantine services and offers space for the development of new online resident management programmes to address the resident entry, management, onsite and exit processes.

Large quarantine operations became more normal as the pandemic progressed, presenting new challenges in COVID‐19 management [26, 27]. The CNR site is situated on 148 acres and consists of approximately 3000 resident rooms, plus staff and site management‐associated infrastructure. Research related to COVID‐19 quarantine environmental factors focuses primarily on infrastructure and air ventilation (particularly in hotel quarantine settings) with recommendations for high disease transmission risk activities to be conducted in open spaces with good ventilation [28, 29, 30].

At CNR, staff were required to interact with residents in the open air (on balconies), and this greatly reduced the risk of disease transmission; however, working outside in tropical conditions meant there was potential for exposure to weather hazards identified as extreme heat, rain and lightning. Staff worked with an ambient temperature of 24.7–33°C with high humidity levels reaching 80+% from November to April in the wet season and 21.6–31.8°C during the dry season. Despite site investment in cooling down areas and restricted time limits to be in PPE in the resident zones, analysis of staff feedback highlighted the combination of time, heat, PPE and distance as being the main logistical challenges. For example, time spent exposed to weather elements whilst in PPE when working with residents in such a large facility. This presented a significant risk for heat stress, further emphasised by the requirement to wear full PPE in a tropical environment and presents another area future services will need to investigate if they have an outdoor facility [31].

A key lesson from the pandemic was the importance of providing a safe environment with effective IPC practices for staff [30, 32]. Despite globally documented COVID‐19 infections among frontline workers, 95% of CNR survey respondents felt safe from contracting the virus at work. At CNR, standard precautions and PPE were implemented, with security stations at zone entries. A buddy system ensured IPC and PPE compliance, although results indicated staff had mixed feelings about security monitoring their practices during donning and doffing. This feedback suggests the need for careful consideration of security officers’ roles in IPC supervision.

All residents entering quarantine were assumed to be a COVID‐19 transmission risk. Residents’ management generally received positive staff feedback, noting that processes included structured arrival and departure information, daily face‐to‐face visits by the health staff, calls/emails/SMS from the Telewellbeing team and access to primary health services with strategies in place for emergencies. All meals were delivered, a click‐and‐collect service was available for residents to order additional food and other items and there was an open‐air balcony that allowed residents outdoor access and to see other residents. This approach was very different from hotel quarantine services, in which residents cited feeling isolated, often with limited access to fresh air [1, 33].

Staff indicated the need for more health service provision to residents, which contradicts CNR's expectation that residents will have a level of self‐care and independence with their health; however, the facility did provide internal referral systems for a comprehensive set of health staff (physiotherapists, occupational therapists, medical officers, mental health nurses and social workers). Residents were encouraged to access external general practices and pharmacies where possible to meet health needs (e.g. seeking online medical consultation with their own general practitioner). It could be argued that residents requiring more access to health services than this primary health care level need to be in a secondary health facility or utilise another model of quarantine such as the medicalised hotel model implemented in Madrid or Special Health Accommodation used in New South Wales, Australia [26, 30].

Staff identified challenges in the quarantine service as falling into two categories, internal factors (site communication, work expectations) and external factors (weather, government legislation). Managing resident and staff behaviour was seen as a mixed challenge. The main challenges staff identified were the weather and heat, as well as less visible issues such as working with inexperienced staff (reflective of rapid recruitment and non‐health workers). These highlight the need for early focus on internal factors and adapting to weather and legislation changes. Overall, staff proved valuable in identifying service strengths and areas for policy improvement. This led to the creation of a pandemic quarantine facility toolbox, now available as an open‐access resource for any situation where quarantine or isolation facilities are required.

### Limitations of this study

Survey information relies on staff recollection and may be subject to recall bias influenced by the positive feelings created by having contributed to an emergency response. The CNR facility exists in the tropical north of Australia, where environmental issues impacted the operation of the facility and featured in the experience of staff at the facility – this may not impact facilities established in other parts of the world. The aspect of pastoral and spiritual care in quarantine for both staff and residents has been explored in other countries; this was not a core part of the CNR model of care and could be further researched for a more holistic approach [34, 35]. The use of non‐health staff as frontline workers in the COVID‐19 pandemic to support health professionals has not been widely researched, and thus there was a limitation in comparing the model used at CNR. This presents an area worth future investigation, as it has great potential to be a model of staffing to boost health workforce capacity in pandemic events.

## CONCLUSION

The staff perceptions of the CNR health model of quarantine provide important insights into the effectiveness of the primary health model of care used at this facility. Dominant areas for improvement were identified in communication with leadership teams, ITMS specific to quarantine needs, staff wellbeing inclusive of education and training, more focus on residential mental health and physical activity and strategies to address environmental challenges faced by a large outdoor infrastructure.

Balancing the needs of residents and staff whilst providing timely and supportive communication, effective infection prevention guides and resources, and training to empower staff with knowledge of disease transmission and management are vital to ensuring facilities and staff are enabled to safely perform their roles. Overall, staff validated the CNR health model of quarantine care, providing evidence to inform quarantine and isolation practices in future public health pandemic responses. This has since contributed to the development of a quarantine resource available for any organisation to use in the event isolation or quarantine of populations is required.

## AUTHOR CONTRIBUTIONS

C*onceptualisation; data curation; software; methodology; investigation; project administration; writing–review and editing; writing–original draft; formal analysis; resources; validation; visualisation*: Angela Sheedy. *Conceptualisation; data curation; formal analysis; investigation; funding acquisition; methodology; supervision; validation; writing–review and editing*: Dianne Stephens. *Conceptualisation; data curation; formal analysis; investigation; project administration; writing–review and editing*: Lisa Vermeulen.

## CONFLICT OF INTEREST STATEMENT

The authors have no conflicts of interest to declare.

## ETHICS STATEMENT

This project received ethical clearance by the Human Research Ethics Committee of the Northern Territory Department of Health and Menzies School of Health Research (HREC Number 2022‐4349).

## Data Availability

Access to the data that support the findings of this study are limited due to ethics agreements; however, all requests will be considered by the corresponding author. Quarantine guidelines, which are representative of the Centre for National Resilience's site functions and strategies (of which this survey contributes), are freely accessible here: https://quarantine‐guide.cdu.edu.au/.
